# Age-Related Deficits of Dual-Task Walking: A Review

**DOI:** 10.1155/2012/131608

**Published:** 2012-07-15

**Authors:** Rainer Beurskens, Otmar Bock

**Affiliations:** Institute of Physiology and Anatomy, German Sport University, Am Sportpark Müngersdorf 6, 50933 Cologne, Germany

## Abstract

This review summarizes our present knowledge about elderly people's problems with walking. We highlight the plastic changes in the brain that allow a partial compensation of these age-related deficits and discuss the associated costs and limitations. Experimental evidence for the crucial role of executive functions and working memory is presented, leading us to the hypothesis that it is difficult for seniors to coordinate two streams of visual information, one related to navigation through visually defined space, and the other to a visually demanding second task. This hypothesis predicts that interventions aimed at the efficiency of visuovisual coordination in the elderly will ameliorate their deficits in dual-task walking.

## 1. Introduction

Accidental falls in old age are an increasing problem in our graying society, and they have received a lot of attention in recent research. About 30% of persons aged 65+ years and about 50% of those aged 85+ years fall at least once a year and the probability of falling again increases after each fall [1–3]. Early research addressed environmental hazards, sensorimotor deficits, and impaired balance as risk factors for accidental falls [4, 5], but more recent work focuses on the role of cognition [6, 7].

According to this recent approach, age-related deficits of locomotion can be partly compensated by cognitive workaround strategies, thus replacing automated sensorimotor processing with effortful higher-order functions. This is a good example of neural plasticity, as it shows that deficits arising in one part of the nervous system can be overcome by engaging another part of that system. Persons with a reduced cognitive capacity have only limited access to this compensation: according to empirical research, they are more likely to walk unsteadily and their risk of falling is higher [8].

Brain plasticity may help overcome the gait problems in old age, but there is a price to pay: cognitive resources allocated to seniors' locomotion are no longer available for other activities while walking, such as obstacle avoidance, navigation along a planned route, watching for pedestrian and vehicular traffic, as well as engaging in gait-unrelated tasks. As a consequence, elderly persons often have larger problems than younger ones to walk and concurrently engage in another activity [6, 7].

## 2. Anatomical Changes in the Human Brain as a Function of Age

Early studies in the 1990s were already able to show age-related changes in the human brain [9–11]. Older people are affected by a general loss of brain mass and a distinctive atrophy of the frontal gray matter, as well as a white matter hyperintensity [12, 13]. Recent fMRI studies showed a degradation of the cerebral cortex as a function of age. Notably, these studies demonstrated a reduction of gray and white matter in the prefrontal cortex [14, 15] and an age-related mass reduction of the frontal lobe [16, 17]. Additionally, a loss of central neurons and associated synaptic connections accrues, which leads to reduced processing speed and a deficit in the ability to handle several processes simultaneously [18]. Some authors state that these structural changes, especially the changes in white matter in the human brain, are caused by an age-related deterioration of the vascular system and the associated reduction in blood flow [19]. Compared with young people, the frontal cortex of people aged 65+ years is reduced by 10–17%, while the temporal, parietal, and occipital cortices only show a reduction of approximately 1%. This selective shrinkage of the brain seems to affect higher-level cognitive functions [14, 20].

 Probably most vulnerable to age-related decay are the so-called “executive functions” [13], that is, cognitive operations that include (a) the planning of strategies for different actions, (b) the monitoring of these actions, (c) adjusting future actions using feedback, alertness, and (d) the inhibition of task-irrelevant information [21]. Another age-dependent cognitive function is working memory, a set of mechanisms involved in the control, regulation, and active maintenance of task-relevant information in both novel and familiar tasks [22–24]. Executive functions and working memory are both thought to reside [25] in the frontal lobes. Recent experiments evaluated the neural activity in prefrontal brain structures while subjects handled tasks involving executive functions or working memory (e.g., the Wisconsin card sorting test, a self-ordered pointing task, or a delayed-response task) and found that these tasks are sensitive to prefrontal dysfunction [19, 26–28]. 

Summing up, the available literature suggests that the anatomical decrease of brain mass, especially in the frontal lobe, contributes to a reduction of cognitive processing capacity with advancing age and thus limits to what extent neural plasticity can compensate for age-related decrements of locomotion.

## 3. The Role of Cognition in Human Locomotion

Human walking, a task that people perform on a daily basis, involves complex processes that require the ongoing integration of visual, proprioceptive, and vestibular sensory information. For instance, joint positions have to be controlled, feedback from the terrain the person is walking on has to be integrated, and the environment the person is moving in needs to be observed. In addition, our everyday life affords numerous situations in which walking must be integrated with another activity, such as watching for traffic or using a mobile phone. This concurrence of locomotion and another activity, termed dual-task walking, has received a lot of attention in recent research [29–33]. The human gait pattern is affected by old age. For example, walking speed and stride length decrease, while lateral sway and stride time variability increase with age [34–36]. Some of these changes are compensatory and are used to stabilize posture, while others are dysfunctional and correlate with the risk of accidental falls [5]. The observed deterioration has been attributed to a variety of causal factors, notably to cognitive decline; indeed, the critical role of cognition is supported by the fact that age-related gait changes are more pronounced in people with cognitive impairment [37, 38] and that they are accentuated under dual-task conditions [6, 39].

The additional tasks utilized in the literature are manifold and range from verbal response and memory tasks to more complex ones such as mathematical tasks or tasks involving visual or motor control. When two or more tasks need to be carried out concurrently, task performance declines at least in one of them. Some studies on dual-task walking found an age-related decrease in dual-task performance when subjects were asked to walk at their preferred walking speed and simultaneously complete another task [39–42], whereas other studies were not able to create this kind of age-related deficit [43–45]. This discrepancy might be due to the utilization of different secondary tasks used in these studies. Some tasks interfere with walking while other tasks do not [46, 47]. A meta-analysis conducted by Al-Yahya showed significant increases in age-related deficits while walking when the secondary task was associated with executive or memory functions, for example, verbal fluency tasks or mental imaging but not when the task added was rather simple, for example, reaction or discrimination tasks [48]. This outcome indicates that the central ability to process walking requirements and cognitive demands simultaneously decreases with age, which might arise from insufficient central processing capabilities in older people [49] or from disorders in the coordination of multiple sensory or motor information [50]. 

### 3.1. Methodological Issues of Previous Studies

A major problem of many studies on age-related decreases in dual-task performance is the inconsistency of methods. Several studies addressing the influence of cognitive functions on locomotion in the elderly have considerable methodological flaws. For instance, some authors observed a decrease in motor performance under dual-task conditions in seniors but did not relate it to the performance of younger subjects and thus could not ascertain the presence of an age-related decrease [26, 51, 52]. Other studies analyzed walking and disregarded the secondary task, or they focused on secondary task and ignored walking; in consequence, they cannot disambiguate changes of dual-task performance from those of task priority [38, 40, 45, 53]. In principle, concurrent tasks can be given equal or different priorities such as to maximize gains or minimize risks [54]; while young people typically prioritize gait [55, 56], older people tend to assign higher priority to the secondary task [30]. As a consequence studies that only evaluate walking will overestimate age-related dual-task deficits, and those that only evaluate the secondary task will underestimate those deficits. 

**Figure 1:**
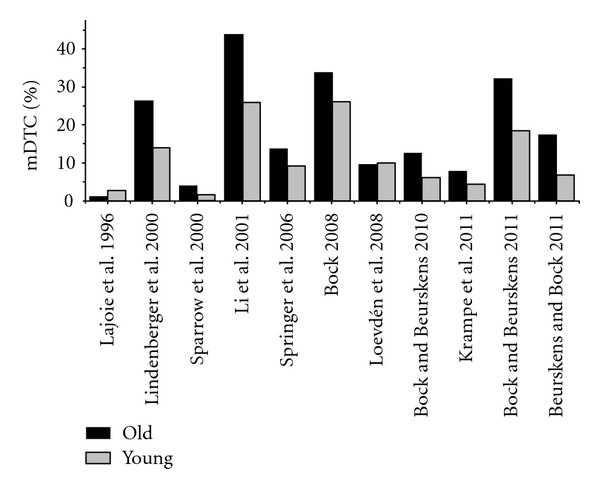
Illustration of mean dual-task costs from different studies evaluating age-related deficits in dual-task walking. Data is calculated from mean values in walking performance and mean values in the secondary tasks used in the studies indicated. Standard errors are not displayed because the data from each individual subject could not be obtained.

In experiments that considered both tasks and both age groups for their analysis, secondary tasks varied in their sensory demands, in their response requirements, or in their cognitive difficulty.  Table 1 shows the mean dual-task costs of young and older subjects taken as examples from recent studies, including the task characteristics of the additional task used.  Figure 1 displays the appropriate dual-task costs derived from the same studies. Dual-task costs were determined according to the following formulas:

(1) DTC=D−SS,

Where (D: dual-task performance; S: single task performance), using the mean values of each age group from the studies indicated.

To express subjects' dual-task ability irrespective of their individual task priorities, we calculated the dual-task costs across both tasks. The formula used is [57, 58]

(2) mDTC=  DTC(task  α)+DTC(task  β)2,

where (task *α*: walking task; task *β*: additional task).

Thus calculated mean dual-task costs show a subject's decrease in performance when completing two tasks simultaneously instead of one task alone. A high DTC value indicates a poorer performance under dual-task conditions compared with single-task conditions. 

As shown in Figure 1, one main difference between all these studies using dual-task paradigms is that different kinds of additional task demands lead to different levels of age-related dual-task costs ranging from 0.99 to 26.0% in young persons and from 2.6 to 44.0% in older aged. Some studies found no deficits of dual-task walking in old age [29, 59, 60], some observed small deficits [41, 44], and others substantial deficits [39, 42, 61–64].

### 3.2. Studies Using Psychomotor Tasks

The wide divergence of age-related deficits in the above studies suggests that the magnitude of deficits is related to demands of the secondary task. For example, Lajoie et al. evaluated the attentional requirements for maintaining posture and walking in eight young and eight older subjects [29]. They combined a sitting, a standing, and a walking task with a verbal response task where reaction times were measured. Subjects were asked to give a verbal response (top) to an auditory stimulus consisting of a 100 Hz tone presented for 50 milliseconds. Observation of the auditory task was conducted either in isolation or in combination with subjects walking along a path at their preferred walking speed. The outcome of this study showed a decline in reaction times and a decrease in walking speed in both age groups. Similar results were observed by Sparrow et al. (2006), who investigated the effects of age on treadmill walking while performing a reaction time task [59]. Subjects had to walk at their preferred walking speed on a treadmill and were instructed to press a hand-held response button as soon as a letter was presented on a monitor in front of them. In single and dual-task condition, the mean walking speed of the young group was significantly faster than the walking speed of the older group, and the reaction times in both groups decreased while treadmill walking. These results suggest that completing psychomotor tasks while walking, irrespective of whether the response is verbal or manual, does not seem to be sensitive or challenging enough to give rise to age-related deficits in dual-task performance.

### 3.3. Studies Using Arithmetic and Memory Tasks

Springer and colleagues evaluated the possible age-related effects of tasks requiring executive functions on human walking [44]. They combined walking with three different tasks: (a) listening to and remembering a simple text, (b) listening to and remembering a complex text, and (c) serial subtracting of seven, starting from 500. Moreover, this study subdivided the older group into “fallers” (one or more fall in the previous six months) and “nonfallers” (no fall in the previous six months). The results identified no age-related effect of dual-task walking when the young group and the older nonfallers were compared. The decrease in walking speed and in secondary task performance was similar. In contrast, when the fallers were compared with the young group, especially with regard to the arithmetic tasks, age-related deficits in dual-task behavior were clearly observable (dual-task costs: old: 14%; young: 9%). The simultaneous processing of arithmetic tasks (subtracting serial 7s) and its cognitive load while walking seems to destabilize the gait of elderly fallers but appears to have no effect in older non-fallers and young people. 

A further approach to evaluate the effects of cognitive load and memory function on dual-task walking was conducted by Loevdén and colleagues [60]. They manipulated working memory load using an n-back task while the subject was treadmill walking. Their paradigm showed that cognitive processes used to solve n-back tasks are not related to the control of human locomotion. The extent of age-related dual-task deficit did not differ between the older and the young people. Just like psychomotor speed, working memory, measured by n-back tasks, does not appear to be a factor in age-related deficits. In addition to that Krampe et al. evaluated the impact of a semantic word fluency task on walking at a fast pace [41]. Subjects were asked to name as many words as possible related to a given category (e.g., “vehicles” or “instruments”). The results indicated a decrease in walking speed for all age groups and this decrease was approximately 3.5% larger in the older group.

### 3.4. Studies Using Visually Demanding Tasks

Recent studies that found a substantial increase of dual-task costs with advancing age were conducted by the groups of Lindenberger et al. [39, 42] and Bock and Beurskens [61–64]. In the studies of the former group, subjects were given a visual memory task where words had to be memorized, and later reproduced, using visuo-spatial imagery. Both studies found higher dual-task costs in the older group compared with younger people, accounted for by the memory task but not by the walking task. The age-related increase of dual-task costs amounted to about 15%, which is higher than that in most other studies. Thus, the additional task used by the Lindenberger group seems to be particularly sensitive to age-related deficits in cognitive performance. Bock and colleagues tested this assumption explicitly by systematically investigating the influence of task characteristics on dual-task walking to find out which aspect of a task is responsible for the development of age-related deficits in dual-task conditions [65]. The authors compared the single- and dual-task gait of young and elderly subjects with eight different combinations of walking and nonwalking tasks, and they found age-related deficits of dual-task walking for some but not for other task combinations. Subjects either had to spell words of 18 to 21 letters, remember sequences of different symbols (triangle, cross, ellipse, etc.), close buttons of nine different shapes and sizes on a jacket, accomplish a reaction time task, walk at maximum speed or on wide/narrow paths, walk on a treadmill, or avoid obstacles that were presented on a treadmill at irregular intervals. All these conditions were administered in single- and in dual-task conditions. Bock and colleagues found that dual-task costs were small in most experiments and did not differ between young and older participants. The only task combination in which dual-task performance was distinctly lower in older than in young subjects were experiments requiring the time-critical processing of visual information (e.g., obstacle avoidance in cooperation with a visual reaction time task). Tasks showing the highest deficits in the elderly combined three main features: (a) the task was conducted on a treadmill, (b) subjects had to avoid obstacles, and (c) the walking and non-walking tasks required continuous visual control. In a second study, Bock explored the effects of the different walking tasks by comparing walking on a treadmill with walking in a hallway [57]. The results from both experiments were similar, overground and treadmill walking had similar effects on age-related dual-task decrements. This outcome leads to the suggestion that walking on a treadmill does not produce deficits in walking as a function of age. 

Furthermore, subjects completed walking with two different additional tasks while avoiding obstacles. One secondary task required visual control (i.e., checking boxes on a sheet of paper) and the other tasks required memory and attention resources (i.e., memorizing pictures). In these task combinations, obstacles did not give rise to any age-related deficits, and only the features of the secondary task led to age-related differences in dual-task performance. An increase in dual-task costs occurred mainly in a task requiring visual processing of information and managing two streams of visual information, one related to the checking task and the other one related to the walking task. These results are indirectly supported by a correlation of postural control and the degree of visual impairment that was found by Jamet and colleagues [66]. Beurskens and Bock extended the outcomes of Bock and colleagues and showed that the visual component of a secondary task has a crucial influence on age-related deficits in dual-task walking [61, 64]. The age-related increase of dual-task costs in their studies amounted to about 9%, which is higher than in most other studies but lower than in Lindenberger's experiments [39, 42]. 

### 3.5. Digression to Studies Using Brief Distractor Tasks Instead of Continuous Ones

In contrast to the methods and tasks used in most of the presented studies, secondary tasks in our everyday life are hardly continuous, and they do not occupy a person for an extended period of time. Usually, the tasks are rather brief, occur rarely and at unpredictable times, for example, stepping over wet spots on the street, watching street signs to work out the right direction or stopping to walk when a car approaches. To consider these kinds of situations, Beurskens and Bock recently compared young and older people's walking behavior after short and unexpected distractions [64]. Eight monitors were arranged at irregular intervals on a straight floor, four to the subject's left, and four to the right. Participants walked the path ten times back and forth at their preferred speed, thus covering a total distance of 400 m and passing 160 times in front of a monitor. On twelve of those passes, they heard the command “left” or “right,” referring to the location of the upcoming monitor. At the same time, a capital letter from the Latin alphabet (such as “G” or “K”) was displayed for 2 seconds on that monitor. Letters were presented mirror-reversed or nonreversed, at a rotation angle of ±60% or ±120° with respect to the vertical. Subjects were instructed to respond to each letter by saying “yes” if it was mirror-reversed, and “no” if it was not reversed. Results showed that brief distractions did not influence younger people's walking but significantly changed older people's walking behavior by increasing step duration and decreasing the amplitude of a step [64]. The age-related decrease in dual-task performance reached up to 14%, which is comparable to Lindenberger's findings with visual imagery. Brief visual distracters, therefore, can be as disruptive for elderly persons as an ongoing demand on visual working memory. 

## 4. Additional Factors Influencing Dual-Task Walking Performance

In addition to age-related cognitive disorders and a deterioration of cognitive functions with advancing age, there are other potential causes of changes in walking behavior and human locomotion. One further aspect often addressed in contemporary literature is the possible correlation of older people's fear of falling with gait changes. Some authors find correlations and state that the development of fear after a fall leads to changes in walking speed, step time, or the appropriate variability, which leads to recurrent falls during the following years [32, 67, 68]. However, other authors find no evidence that preceding falls increase the occurrence of subsequent falls [69, 70] and cannot show that the fear of falling is associated with a reduction of physical activity for safety reasons [71]. In fact, the correlation of psychological functions and human walking is a multidimensional construct where several aspects, ranging from fear of falling, education, physical activity and stress, have to be taken into consideration. 

Another possible confounding factor in this area of research is the role of eye movements. Subjects typically focus both their gaze [72, 73] and their attention on the goal of their activities [74, 75], which indicates that attention, eye and body movements are all closely interlinked. However, oculomotor behavior changes with advancing age: the latency and duration of saccades increase while their accuracy decreases, thus necessitating more corrective and re-fixation saccades [76–78]. Such deficits could complicate the navigation through visually defined space and its integration with another visual task, and thus contribute to impaired dual-task walking.

Yet another factor to be considered is the age-related shrinkage of the attention window, which can be observed not only with cognitive tasks [79, 80] but also with manual ones [62]: bimanual tracking deteriorates with display distance in older, but not in young subjects. However, the magnitude of this deterioration in a given elderly person is not related to that person's dual-task costs when walking with a concurrent visuospatial task (*R* ^²^ = 0.04; *P* < 0.05), which suggests that peripheral visual attention may not be an important factor in dual-task walking.

## 5. Conclusions and Outlook

When reviewing contemporary research on dual-task walking, motor control, and the role of cognitive functions, the topics of executive functions, and the role of the human frontal lobe have often been addressed. Many studies associate the occurrence of dual-task deficits in the elderly while walking with the well-known decay of prefrontal cortical circuitry, the loss of prefrontal brain mass, and the associated deterioration of executive functions in old age [16, 20, 81]. The current review shows that such deficits are observable mainly with nonwalking tasks requiring substantial visual processing, and thus with the coordination of two independent visual streams of information. It is quite conceivable that this coordination depends critically on higher-level cognitive functions, such as executive functions and working memory, while peripheral visual attention does not seem to play a predominant role. This would fit well with Norman and Shallice's concept of executive control [82], according to which shifts of attention from one task to another take place in a high-level “supervisory attentional system.” 

That being said, we must add that not all age-related deficits of dual-task walking can be explained by interference between two visual streams. Deficits have also been reported for tasks without a visual component, although they were much smaller than those for tasks having a visual demand [29, 41, 59] or were limited to very old subjects (75+ years). It, therefore, appears that interference is not necessarily confined to the visual modality but rather can be intermodal, and increasingly so in very old age.

Summing up, we suggest that neural plasticity can partly compensate for age-related deficits of walking: it supplements deteriorated sensorimotor processes by cognitive processes. However, this compensation reduces the cognitive capacity available for concurrent tasks, and is limited by the age-related decay of the prefrontal cortex.

## Figures and Tables

**Table 1:** Summary of main characteristics of the studies included in this review.

Reference	Groups	mDTC (%)		Dual-tasks		Outcome	Significance
		Young	Older	Walking task	Secondary task
Lajoie et al. 1996 [29]	young: *n* = 8 (26.0 ± 4.2 y) old: *n* = 8 (71.0 ± 4.2 y)	0.99	2.63	normal pace walking	manual task: reaction time	velocity (m/s)	n.s.
Lindenberger et al. 2000 [39]	young: *n* = 47 (24.0 ± 3.2 y) middle: *n* = 45 (45.0 ± 3.3 y) old: *n* = 48 (65.0 ± 3.1 y)	14.0	26.5	normal pace walking (straight and curved narrow path)	visuospatial decision task	velocity (m/s)	∗∗
Sparrow et al. 2006 [59]	young: *n* = 10 (26.3 y) old: *n* = 10 (71.1 y)	1.51	3.94	normal pace walking on a treadmill	manual task: reaction time	velocity (m/s)	n.s.
Li et al. 2001 [42]	young: *n* = 37 (25.1 ± 2.7 y) old: *n* = 40 (65.6 ± 3.9 y)	26.0	44.0	normal pace walking (wide path)	visuospatial decision task	velocity (m/s)	∗∗∗
Springer et al. 2006 [44]	young: *n* = 19 (29.4 ± 4.4 y) fallers: *n* = 17 (76.1 ± 4.8 y) non-fallers: *n* = 24 (71.0 ± 5.9 y)	9.25	13.61	normal pace walking	arithmetic task: counting backwards by 7	velocity (m/s)	n.s.
Bock 2008 [57]	young: *n* = 18 (24.3 ± 3.5 y) old: *n* = 15 (67.2 ± 3.6 y)	26.0	34.0	normal pace walking (narrow path)	manual task: checking	velocity (m/s)	∗∗∗
Loevdén et al. 2008 [60]	young: *n* = 32 (25.0 ± 2.9 y) old: *n* = 32 (73.6 ± 2.9 y)	9.93	9.64	slow and normal pace walking (wide path)	arithmetic task: counting backwards by 1–4	velocity SD (m/s)	n.s.
Bock and Beurskens 2011 [63]	young: *n* = 15 (25.4 ± 2.9 y) old: *n* = 15 (69.2 ± 4.7 y)	6.01	12.46	normal pace walking (narrow path)	manual task: checking	velocity (m/s)	∗∗∗
Krampe et al. 2011 [41]	young: *n* = 30 (24.3 ± 2.2 y) old: *n* = 30 (64.2 ± 2.4 y)	4.25	7.75	normal pace walking (narrow path)	verbal task: word fluency	missteps (%)	∗
Bock and Beurskens 2011 [64]	young: *n* = 12 (25.6 ± 2.8 y) old: *n* = 12 (68.1 ± 4.2 y)	18.38	32.28	normal pace walking (wide path)	visuospatial decision task	step duration (s)	∗∗∗
Beurskens and Bock 2011 [61]	young: *n* = 14 (22.0 ± 2.1 y) old: *n* = 14 (69.1 ± 3.4 y)	6.84	17.41	normal pace walking (narrow path)	visuospatial reading task	velocity (m/s)	∗
